# The Current State of Primary Clinical Spine Disease Research in Sub-Saharan Africa — A Scoping Review

**DOI:** 10.1177/21925682261498667

**Published:** 2026-10-26

**Authors:** Charlotte F. Wahle, Maithily Diaz, Michael Oravic, Shravya Kakulamarri, Babapelumi Adejuyigbe, Chellandra Samuels, Chibuikem Anthony Ikwuegbuenyi, Romani Sabas, Drew N. Wright, Saam Morshed, Ashraf N. El Naga, Roger Härtl, Hamisi Shabani, David Shearer, David Gendelberg, East Africa Spine Trauma Research Group

**Affiliations:** 1 Institute for Global Orthopaedics and Traumatology, UCSF, San Francisco, CA, United States; 2 University of California, San Francisco School of Medicine, San Francisco, CA, United States; 3 Geisinger School of Medicine, Scranton, PA, United States; 4 Department of Neurological Surgery, Och Spine at New York Presbyterian Hospital, 12295 Weill Cornell Medicine, United States; 5 296671 Department of Neurosurgery, Muhimbili Orthopaedic Institute, Dar es Salaam, Tanzania; 6 Samuel J. Wood Library, C.V. Starr, 189446 Weill Cornell Medical College Biomedical Information Center, New York, NY, United States; 7 Department of Orthopaedic Surgery, University of California, San Francisco, United States

**Keywords:** trauma, decompression, burst fracture, adolescent, degenerative, spinal cord injury, tumors, orthopaedic

## Abstract

**Study Design:**

Scoping Review.

**Objective:**

Spine research is resource-intensive and thus often performed in high-income countries. Consequently, findings may not be generalizable to low-resource areas. This study aimed to assess the current state of primary clinical spine disease research in Sub-Saharan Africa.

**Methods:**

All spine studies published in English or French in Sub-Saharan Africa in the past 50 years with at least ten patients were included. A study quality variable was created that identified randomized studies, prospective studies, and studies that include a control group as having a higher level of scientific rigor.

**Results:**

A total of 154 spine studies have been published from at least 26 countries since 1975 (some included multiple). There was a significant increase in research productivity after 2005 (p<0.001). The highest number of publications came from Nigeria with 52 (34%), followed by Tanzania (25 papers, 16%) and Ethiopia (12 papers, 8%).

Nearly half of studies involved collaborative partnerships. On univariate analysis, lower-income countries were more likely to engage in collaboration with other countries (OR 3.2, 95% CI: 1.2-9.8) and to receive external funding (OR 7.0, 95% CI: 2.3-20.4). Further, collaborative studies were found to produce higher quality academic publication (OR 2.0, 95% CI: 0.94-4.2).

**Conclusion:**

There is a relative paucity of spine literature from Sub-Saharan Africa. Higher research quality was associated with collaborative engagement. These results support the development of global health partnerships that may foster high-quality research aimed at addressing the burden of spine disease in Africa.

## Introduction

The global public health burden of spine injuries is high, surpassing that of many infectious diseases, with a disproportionate number of traumatic spine injuries occurring in low and middle income countries (LMICs).^1,2^ Spine pathology research can be resource intensive leading to it being more often performed in high-income countries (HICs).^3,4^ However, patients treated in LMICs face unique challenges when it comes to healthcare access and resource availability. Thus, the existing literature, conducted largely in HICs may not be generalizable to these populations.

Spine research has experienced significant growth over recent decades. Between 2004 and 2013, the number of published articles in spine surgery grew 1.9-fold — signaling an upward trend in research activity.^4^ However, the majority of this research originated in HICs (particularly North America, East Asia, and Western Europe) where adequate funding, advanced healthcare infrastructure, and specialized medical training provide the foundation for such studies.^3,5^ In fact, between 2004 and 2013, Wei et al. identified that the United States, Japan, and China alone were responsible for more than 50% of all articles in the field of spine surgery.^4^ This imbalance underscores the persistent disparity in the global landscape of spine research, where financial and infrastructural constraints continue to limit the capacity for research in LMICs. This is despite the pressing need for generalizable, high-quality evidence to guide treatment decisions.

The major barriers to performing high-quality research in LMICs include resource availability, infrastructure, and education. As such, there has been growing interest in HIC-LMIC collaborations which may help supply necessary financial support, strengthen existing research infrastructure and provide mutual learning opportunities. These collaborations are becoming more common. This is a promising trend given the fact that recent literature has demonstrated that previous HIC-LMIC partnerships resulted in increased research capacity for both parties as well as policy and practice reform.^6^ Moreover, capacity building for research, with the goal of building towards independent, sustainable initiatives, empowers local community members to lead investigations which aim to answer locally relevant questions that will best serve their communities.^7^ In order to be successful as a researcher, effective training and mentorship from someone with experience is essential. However, research practices are often not included in much of global medical education curricula.^8^ When trust and reciprocity are embedded within these collaborations, both LMICs and HICs benefit, creating a foundation for sustainable and impactful research efforts in global health.

While research efforts in spine surgery and collaborations have expanded significantly in recent decades, the strength of this progress remains unclear. Therefore, the aims of this study were to assess the current state of the literature regarding spine-related publication in sub-Saharan Africa as well as to evaluate the degree to which collaborative partnerships may serve to promote the conduct of high-quality spine research in low resource settings.

## Methods

A scoping review of the literature was performed according to Preferred Reporting Items for Systematic Reviews and Meta-Analyses (PRISMA) guidelines. The aim of the search was to evaluate the current state of the literature with respect to spine-related research that was based in sub-Saharan Africa. The search was conducted across three academic databases, including PubMed, Ovid Medline and Embase. A medical librarian assisted in the development of MeSH terms for the study to capture the breadth of spine pathologies (Appendix 1).

All articles that pertained to primary spine pathology research conducted in Sub-Saharan Africa that were published in either English or French over a fifty-year period from 1975-2024 were included in the study. Spine pathology was defined as any acute or chronic spine trauma injury or disease of the cervical, lumbar, thoracic, sacral or spinal cord. Research pertaining to both congenital and acquired disease of the spine in both children and adults were included. Exclusion criteria included studies with fewer than 10 patients, studies taking place in Northern Africa or South Africa, and studies published in a language other than English or French. South Africa was excluded because its research infrastructure and overall health system capacity are outliers from other Sub-Saharan countries, more closely resembling an upper-middle-income settings. Including it would have reduced comparability across countries given the aims of the review. Non-primary research articles were also excluded, including narrative reviews, commentaries and editorials.

Five independent researchers (MD, MO, CAI, BA, RS) participated in screening the articles — first by titles, followed by abstracts and subsequent full-text review. Each text was reviewed by a minimum of two independent reviewers. If there was inter-reviewer disagreement between the two initial reviewers, a third reviewer evaluated the study (CW). Any remaining questions or uncertainty was discussed with the senior author, a spine fellowship–trained orthopaedic surgeon (DG).

Following full-text screening, data was extracted and compiled from the articles. Study characteristics were collected including study design, location, author nationality, collaborative partnerships and funding source. Clinical information was also collected including patient demographics, anatomic location of the pathology, etiology of pathology**,** type of surgical or non-surgical intervention, and outcome measures. Descriptive statistics were performed as well as chi-squared analysis for categorical variables. Significance was set at *p*<0.05. All statistical analysis was performed using STATA V18 (StataCorp, College Station, TX).

## Results

A total of 1895 studies resulted from search criteria across 3 databases (Figure 1:). Duplicates were removed — resulting in 1433 abstracts for screening. Following this, 249 full texts were reviewed, with 154 studies meeting final inclusion criteria.

**Figure 1:**
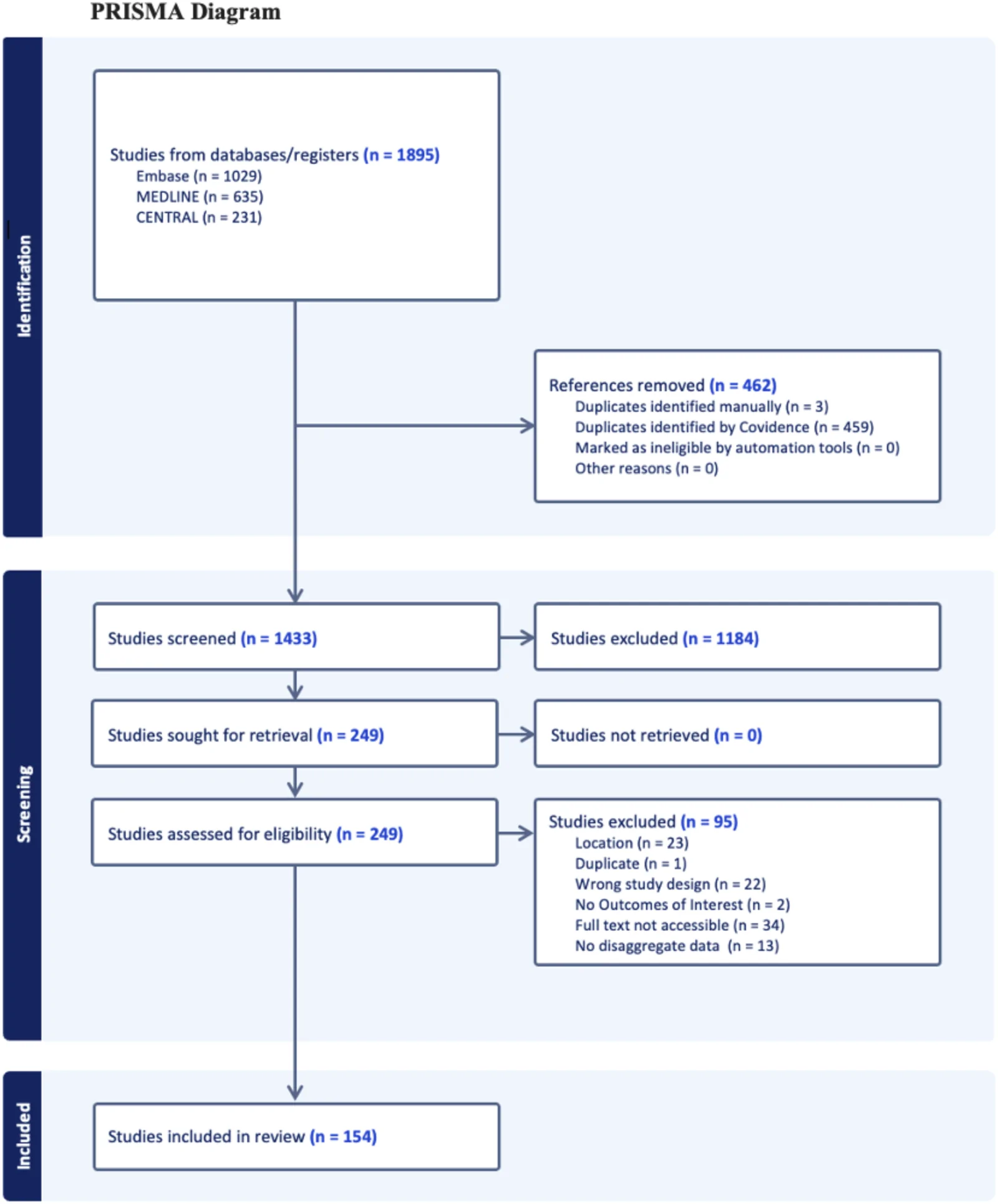
PRISMA Diagram

### Geographic locations

A total of 154 spine studies have been published out of at least 26 countries since the year 1975 (Figure 2:). Some studies included multiple African countries. The number of spine studies increased over time, with a significant increase beginning in 2005 (p<0.001) (Figure 3:). Over half (58%) of the studies came from just three countries. The highest number of publications came from Nigeria with 52 (34%), followed by Tanzania (25 papers, 16%) and Ethiopia (12 papers, 8%) (Table 1). A total of 21 studies (14%) included patients from multiple treatment centers within sub-Saharan Africa.

**Figure 2:**
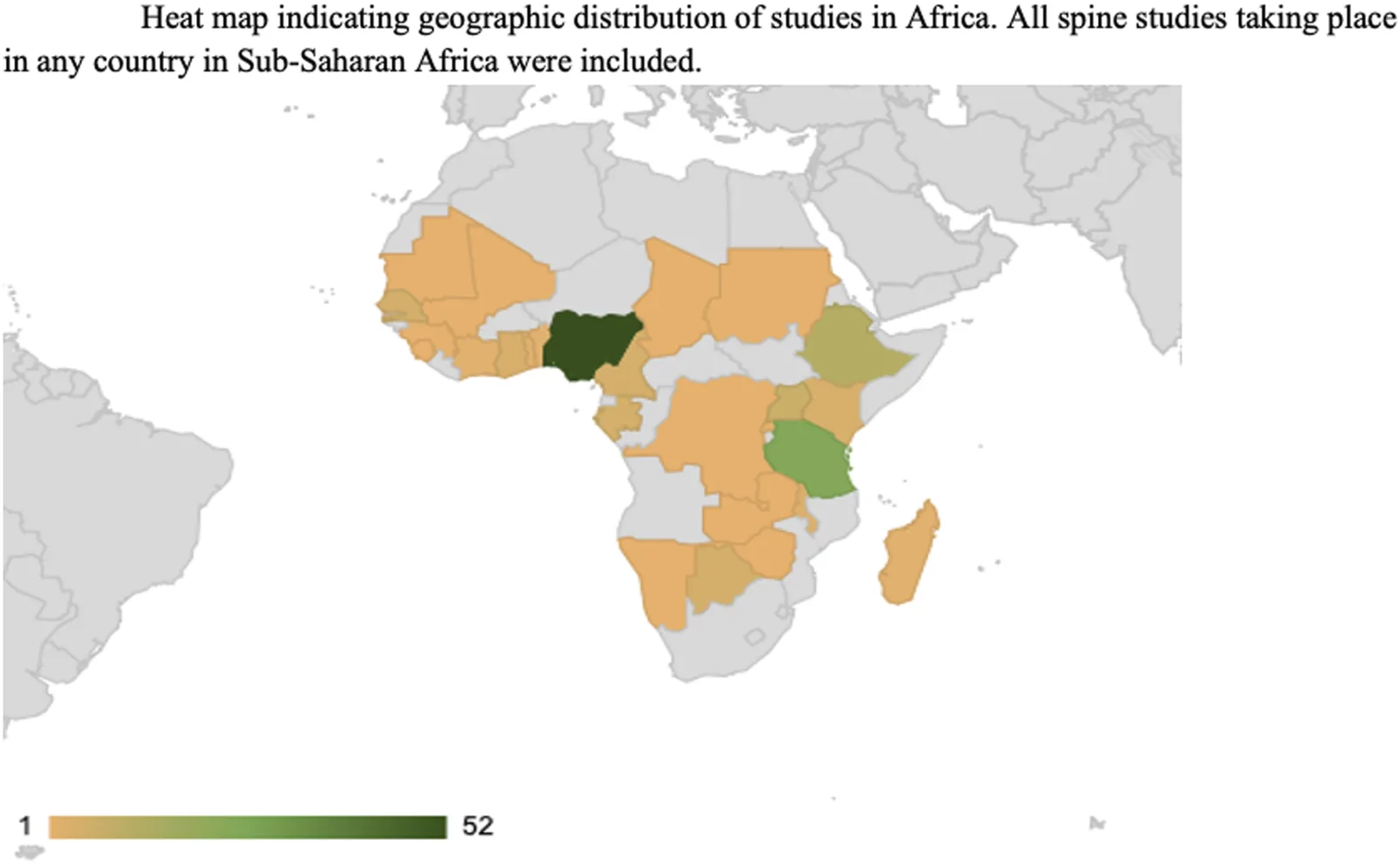
Heat map indicating geographic distribution of studies in Africa. All spine studies taking place in any country in Sub-Saharan Africa were included

**Figure 3:**
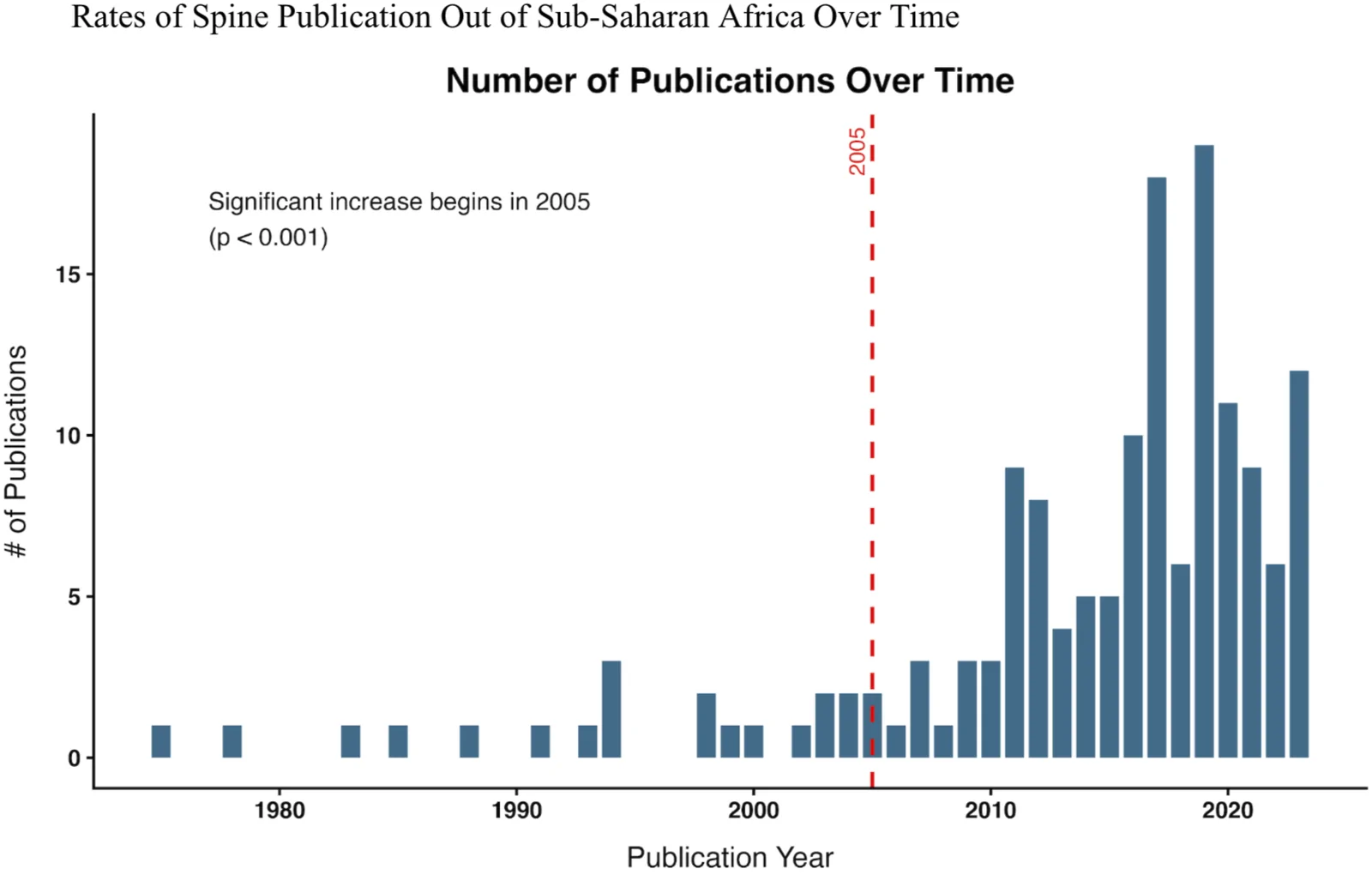
Rates of Spine Publication Out of Sub-Saharan Africa Over Time

**Table 1.** Spine publications by (a) country and (b) region

Most Published Countries	
Country	Publications (N)
Nigeria	52
Tanzania	25
Ethiopia	12
Uganda	7
Botswana	5
Cameroon	5
Gabon	5
Senegal	5

Regional Distribution	
Region	Publications (N)
West Africa	72
East Africa	56
Central Africa	12
Southern Africa	10
Multiple Sub-Saharan African Countries	4

### Study Details

The authors of the published studies came from a variety of specialties across medicine and public health. The three most commonly represented specialty were neurosurgery (35%) followed by orthopaedic surgery (19%) and then physical medicine and rehabilitation (8%).

The vast majority of the studies were retrospective cohort studies (62%) followed by prospective cohort studies at 26% (Table 2). Three of the studies (2%) included a control group. There was a single randomized controlled trial. The most common study purpose was epidemiologic (103 studies, 67%), followed by therapeutic (49, 32%) studies. Other less common study types included qualitative (9, 6%), diagnostic (11, 7%), and economic analysis (8, 5%).

**Table 2.** Study Characteristics

Category	N	%*
Articles	154	​
Year Range		
2015 - 2024	96	62.34
2005 - 2014	39	25.32
1995 - 2004	9	5.84
1985 - 1994	7	4.55
1975 - 1984	3	1.95
Study Classification		
Retrospective	93	61.59
Prospective	39	25.83
Review	15	9.93
Cross-Sectional	2	1.32
Randomized Controlled Trial	1	0.66
Case Series	1	0.66
Study Population		
Mixed	93	60.39
Adult	57	37.01
Pediatric	2	1.30
Unspecified	2	1.30
Funded Study		
Unspecified	111	72.08
Yes	24	15.69
No	19	12.42
Multi-Center Patient Population		
No	133	86.36
Yes	21	13.64
Collaborative Study		
No	80	51.95
Yes	74	48.05
Control Group Used		
No	151	98.05
Yes	3	1.95
Randomized Study		
No	152	98.70
Yes	2	1.30

Many studies assessed multiple spine regions, with the spinal cord being the most commonly evaluated (106, 69%), followed by the cervical spine (103, 67%), lumbar spine (87, 56%), and thoracic spine (83, 54%). Among the studies included in the review, 75% discussed trauma as a spine disease etiology. The next most common etiologies were degenerative (23%) and infectious (22%), which were discussed in a similar number of studies. Studies reporting on deformity and congenital causes of spine disease made up 10% of total studies, respectively. The three most commonly reported outcome measures included functional outcomes (75%), mortality (62%), and infection (42%). Reoperation was an outcome reported in 5% of studies. More than half (61%) of the included studies consisted of mixed adult and pediatric study populations, while 36% studied adults only and 1% studied children only (2% did not specify).

### International Collaboration

Studies were also classified with respect to the socioeconomic status of the country in which they were published (Table 3). The most common socioeconomic status was lower middle-income with 118/154 (77%) countries. Nearly half of the studies (74/154, 48%) involved collaboration with another site or country. The most common collaborating countries included the United States, who collaborated on 34/154 studies (22%), followed by Sweden (8, 5%) and Spain (7, 5%). A total of 24 studies (16%) disclosed external funding sources, however a major proportion of the studies did not specify their funding status.

**Table 3.** Spine Publications by World Bank Classification

Category	N	%
LICs	25	16.23
Ethiopia	12	7.79
Malawi	3	1.95
Madagascar	2	1.30
Rwanda	2	1.30
Chad	1	0.65
Democratic Republic of Congo	1	0.65
Guinea	1	0.65
Mali	1	0.65
Sierra Leone	1	0.65
Sudan	1	0.65
LMICs	112	72.73
Nigeria	52	33.77
Tanzania	25	16.23
Uganda	7	4.55
Cameroon	5	3.25
Senegal	5	3.25
Ghana	4	2.60
Kenya	4	2.60
Benin	2	1.30
Côte d’Ivoire	2	1.30
Togo	2	1.30
Mauritania	1	0.65
Zimbabwe*	3	1.95
UMICs	12	7.79
Botswana	5	3.25
Gabon	5	3.25
Namibia	1	0.65
Zambia	1	0.65
Multiple sub-Saharan African countries*	5	3.25

Percentage of total articles (n = 154).

LICs = low-income countries; LMICs = lower-middle-income countries; UMICs = upper-middle-income countries; HICs = high-income countries.

*These studies reported findings from more than one sub-Saharan African country. Multicountry studies (n = 5) were counted once per study and not apportioned across included countries.

**These studies include findings from Rhodesia, the former name of Zimbabwe.

Overall, forty-eight (31%) studies were of a higher level of study quality per the criteria described above. On univariate analysis, lower income countries were both more likely to engage in collaboration with other countries (OR 3.2, 95% CI: 1.2-9.8) and to receive external funding (OR 7.0, 95% CI: 2.3-20.4). Further, collaborative studies were found to be more likely to produce academic publication at a higher level of study quality (OR 2.0, 95% CI: 0.94-4.2).

## Discussion

We present a scoping review of all spine-related research taking place in Sub-Saharan Africa over the past 50 years (1975-2025). In summary, a total of 154 spine studies have been published out of 32 countries. Over 50% of the published studies came from just three African countries: Nigeria, Tanzania and Ethiopia. There was also a high prevalence of collaboration between African countries and an additional higher-income country outside of Africa. Low-income countries were significantly more likely to engage in these collaborations. Collaborative studies were also found to significantly increase the likelihood of higher quality academic publications.

### Challenges in Spine Research

Given the high proportion of life-threatening injuries, significant resource demands, and highly specialized care required, the execution of high-quality spine research can be challenging without robust resources. In the present state, the vast majority of spine research being conducted takes place in HICs. Meanwhile, the World Health Organization (WHO) Global Burden of Disease study found that spine disorders are a significant source of substantial disability in low and middle-income countries.^9,10^ This highlights a discrepancy that is often referred to as the “10/90 gap,” whereby approximately 10% of the world’s medical research funding is addressing the 90% of the population residing in LMICs.^11^ As such, this creates issues of generalizability of HIC research when it comes to the implementation of evidence-based practice in LMICs.^9,12-14^ There are several barriers to the conduct of high-quality spine research in LMICs. First, there is the challenge of research methodology education. Much of the global surgical education research does not explicitly cover research methodology, as the education often prioritizes practical, technical surgical skills to help address the high trauma burden and limited access to surgical education.^15-18^ However, in recent years, research education has been increasingly recognized as a critical component of capacity building in LMICs, prompting calls for its prioritization.^15,19^ Second, there is the challenge of access to financial resources.^9,13,14^ The cost of spine research can be prohibitive, especially in LMICs. Given that a high proportion of spine pathologies in LMICs consist of traumatic injuries^20-22^ proper outcomes research often requires expensive implants and other operative equipment including advanced imaging.^23,24^ Further, even when implants are donated, patients in LMICs are often uninsured and asked to pay surgeon fees upfront, leading to substantial delays in surgery.^25-27^ Thus, the conduct of spine research in LMICs can be challenging. The two most central barriers to entry can be generally grouped into education and funding — creating a ripe opportunity for mutually beneficial collaboration with HICs.

### International Academic Partnerships

International research collaborations across a wide variety of medical disciplines have been growing in prevalence over the past several decades.^12-14,28^ In the present study, a significant increase was observed in spine research publication out of Sub-Saharan Africa, tripling between 2005-2015. There has been continued significant growth in recent years, doubling again between 2015-2024. In another recent systematic review looking at international orthopaedics as a whole, Flores et al reported that international orthopaedic academic partnerships had nearly doubled over the study period from 2017-2022.^28^ This comes in the wake of the Lancet Commission on Global Surgery 2030 calling for the need for unified commitment to research collaboration in order to ensure safe and affordable access to surgical care.^29,30^ In recent years, there has been an increasing emphasis in shifting towards more equitable, mutually beneficial research partnerships between HICs and LMICs.^12,14,28,31^ This model has proven to be highly successful in a wide variety of geographic locations.^12,14,15,28,31,32^ Moreover, the present study found a direct relationship between international collaborations and study quality. Of the 154 included studies, it was found that approximately one third of the included studies (48/154) were deemed to be of “higher quality.” This designation was defined as randomized controlled trials, prospective studies, or those which included a control group. This is consistent with a prior scoping review by Ibrahim et al in pediatric orthopaedics which found that collaborative papers were more likely to reach a higher level of evidence than non-collaborative studies.^14^ Similarly, in another study by Breugelmans et al, it was found that publishing in open access journals and HIC-LMIC international collaboration resulted in a significant increase in research impact, as measured by a boost in citation rates.^33^ Flores et al reached a similar conclusion in the orthopaedics literature in 2024, reporting that HIC-LMIC collaborative studies which were published in open access journals had a significantly higher Journal Citation Indicator.^28^ Finally, in another study by Morshed et al, a similar relationship was found between HIC-LMIC academic collaborations and a higher level of evidence.^31^ Thus, HIC-LMIC partnerships can certainly offer both sides benefit in producing high-quality research that is generalizable to LMICs, meanwhile helping build-up research capacity at LMIC institutions to help them move towards research independence.

### Building ethical collaborations

While there is a clear benefit which can be derived from mutually beneficial, equitable collaborations, this requires reciprocal effort and communication to ensure mutual understanding of research aims and resource availability. It is critical that both sides discuss their relative goals for the relationship and engage in frequent discussions to ensure that the relationship remains equitable and without unintended power dynamics on either side.^13^ Several prior works have discussed best practices for approaching these inter-country collaborations, particularly those that exist between higher and lower-income countries.^9,12,13^ In a recent 2024 study evaluating the critical elements of international academic partnerships through a modified Delphi approach, 54 items reached consensus as critical elements of a collaboration. Several of the most common themes included those which referred to collaboration and communication, bidirectional data exchange, collaborative project development, bidirectional authorship representation, discussion of shared goals, bidirectional feedback and discussion of challenges.^13^ Another theme to consider in practically conducting HIC-LMIC collaborative research is local resource barriers, ensuring that there is appropriate, safe equipment present in-country to conduct the research.^9,12^ This consideration not only includes patient-specific study equipment, but also infrastructure for data collection and entry, such as a stable internet connection or a mechanism of collecting data offline.^12^ It is also important to consider potential barriers in recruitment and retention, such as translating consent forms into lay terms in the local language, gathering contact information from the patients’ close contacts, and covering costs of follow-up imaging and travel to visits.^9,12^ Additionally, it is imperative to maintain consistent lines of communication in-country, with a mutually easy to use mode of communication, such as WhatsApp or Messenger (Meta).^12^ Following the conclusion of a project, authorship should be granted to investigators from both sides of the collaboration as well as offered opportunities to present at academic conferences.^12,13^ In addition to aiding in capacity building, this helps promote dissemination to target audiences both locally and internationally, which may help expand the work’s impact. Thus, while the results of the present study indicate the potential benefits of HIC-LIC collaborations, it is important to approach these relationships with thoughtful communication and transparency on both sides.

### Limitations

There are several limitations to this study. First, as with any robust review of the literature, there is the possibility of missed articles which were not indexed in the included databases. However, with the inclusion of three robust databases (Embase, MEDLINE, and CENTRAL), it is the belief of the authors that this risk has been minimized to the extent to which it is feasible. Additionally, the study included all articles published in either English or French as these are two of the primary languages used in the academic literature in Sub-Saharan Africa. However, it is possible that articles were missed if they were not published in one of these two languages. Additionally, for the purposes of socioeconomic analysis, countries were classified according to WorldBank income classifications.^34^ It is important to note that this classification is limited as it does not consider individual and community-level variation within a given country. There may be considerable variation within a given country, and as such, this information would not be captured. With that said, this classification has been used to classify socioeconomic status at a country level in several prior studies.^28,35^ Finally, the “higher quality” proxy used in this study is limited and not a validated measure, however given the substantial heterogeneity across studies, it provided a practical way of stratifying the rigor of experimental design. The heterogeneity across studies also limited the authors’ abilities to perform meta-inference, which is another challenge that the proxy was intended to address and is appropriate within the context of a scoping review.

## Conclusion

There is a relative paucity of spine literature from Sub-Saharan Africa. Higher research quality was associated with engagement in collaboration. These results support the development of global health partnerships that may foster highquality research aimed to address the considerable burden of spine disease in Africa.

## Supplemental Material

Supplemental Material - The Current State of Primary Clinical Spine Disease Research in Sub-Saharan Africa — A Scoping Review Supplemental Material for The Current State of Primary Clinical Spine Disease Research in Sub-Saharan Africa — A Scoping Review by Charlotte F. Wahle, Maithily Diaz, Michael Oravic, Shravya Kakulamarri, Babapelumi Adejuyigbe, Chellandra Samuels, Chibuikem Anthony Ikwuegbuenyi, Romani Sabas, Drew N. Wright, Saam Morshed, Ashraf N. El Naga, Roger Härtl, Hamisi Shabani, David Shearer, David Gendelberg, East Africa Spine Trauma Research Group in Global Spine Journal

## Data Availability

All data discussed in this study is derived from previously published articles, all of which are cited in the manuscript. The compiled data extraction sheet generated during the systematic review process is available from the corresponding author upon request.*
