# Supplementary material for: The Current State of Primary Clinical Spine Disease Research in Sub-Saharan Africa — A Scoping Review
Source: Global Spine J. 2026 Oct 26:21925682261498667. Online ahead of print. doi: 10.1177/21925682261498667 (PMC13710443; doi:10.1177/21925682261498667)
Supplement: Supplementary file 1 [file sj-pdf-1-gsj-10.1177_21925682261498667.pdf]

Study (Author, Year)	Country	Collaborating Countries	Study Design	Topic	Population, Sample Size (n)	Collaboration Type	Funding Source
Oke 2012	Nigeria		Prospective	physiotherapy measures on musculoskeletal parameters	MIxed, 15	Academic	Not reported
Mahomed 1975	Rhodesia		Retrospective	paraplegia	Mixed, 100	Academic	Not reported
Anonymous 1978	Rhodesia, Hong Kong	Rhodesia, Hong Kong	Randomized Controlled Trial (RCT)	anterior spinal fusion for tuberculosis of the spine	Mixed, 280	Multicenter/ International	Not reported
Iwegbu 1983	Nigeria		Retrospective	traumatic paraplegia	Mixed, 48	Academic	Not reported
Okonkwo 1988	Nigeria		Retrospective	spinal cord injuries	Mixed, 72	Academic	Not reported
Loembe 1991	Gabon		Retrospective	spinal cord injuries	Mixed, 122	Academic	Not reported
Chapp-Jumbo 2004	Nigeria	Saudi Arabia	Retrospective	acute spine injuries	Mixed, 1393	Multicenter/ International	Not reported
Belachew 2007	Ethiopia		Systematic review	myelopathy in Africa	Mixed	Academic	Not reported
Zellner 2010	Tanzania	Austria, Germany	Retrospective	spinal lesions in urban and rural areas	Mixed, 1163	Multicenter/ International	Not reported
Cadotte 2010	Ethiopia	Canada, United States	Retrospective	consequence of delayed treatment	Mixed, 107	Multicenter/ International	Not reported
Bayray 2012	Ethiopia		Retrospective	osteoporotic fractures	Adult, 2294	Academic	Yes
Diallo 2014	Mali	Senegal	Prospective	epidemiology and outcomes of falls from trees	Mixed, 73	Academic	Not reported
Moshi 2015	Tanzania	South Africa	Cross-sectional	QoL with spinal cord injury in the rural area	Mixed, 15	Multicenter/ International	Yes
Faduyile 2017	Nigeria		Retrospective	demographics, cause of death and anatomical pattern of injuries	Mixed, 142	Academic	No
Eaton 2017	Malawi	United States	Retrospective	mortality probability in trauma based on anatomic location of injury.	Mixed, 8569	Multicenter/ International	Yes
Abdelgadir 2017	Uganda	USA (Duke University, UCLA)	Retrospective	epidemiology of neurosurgical conditions, establish neurosurgical needs	Mixed, 1854	Multicenter/ International	Yes
Musubire 2018	Uganda	United States	Prospective	non-traumatic SCI	Adult, 103	Multicenter/	Yes

						International	
Leidinger 2018	Tanzania	United States	Retrospective	spine trauma costs and outcomes	Adult, 180	Multicenter/ International	Yes
Doleagbenou 2019	Togo		Retrospective	present neurological practice in low resource hospital	Mixed, 192	Academic	Not reported
Gaye 2019	Senegal	Mauritania	Retrospective	NMO spectrum disorders	Mixed, 16	Multicenter/ International	Not reported
Boushab 2019	Mauritania	France	Retrospective	spinal tuberculosis	Mixed, 115	Multicenter/ International	Not reported
Chikani 2019	Nigeria		Retrospective	spine surgery in a low-income country	Mixed, 463	Academic	Not reported
Ibrahim 2020	Sudan		Retrospective	bio-marker status, outcome and correlation with AQP4	Adult, 31	Academic	Yes
Lessing 2020	Tanzania	United States, Spain	Retrospective	pressure ulcer development in low-cost environment	Mixed, 267	Multicenter/ International	Not reported
Lessing 2020	Tanzania	United States	Retrospective	operative decision-making, neurologic outcomes	Mixed, 97	Multicenter/ International	No
Eyenga 2020	Cameroon		Retrospective	carotid tubercle, traumatic cervical spine injury.	Adult, 34	Academic	Not reported
Romuald 2020	Côte d'Ivoire	United Kingdom, France	Retrospective	prognostic factors for survival in spinal cord compression	Adult, 60	Multicenter/ International	Not reported
Tang 2022	Rwanda	United States	Prospective	neurotrauma outcomes	Adult, 216	Multicenter/ International	Yes
Moshi 2022	Tanzania	Sweden	Retrospective	traumatic spinal cord injury	Mixed, 288	Multicenter/ International	Yes
Oyediran 2022	Nigeria		Retrospective	spinal cord injury	Mixed, 132	Academic	Not reported
Oyemolade 2022	Nigeria		Prospective	pediatric traumatic spinal cord injury	Pediatric, 20	Academic	Not reported
Mugisa 2023	Uganda		Cross-sectional	to assess the perceived quality of life of patients with traumatic spinal cord injuries	Adult, 103	Academic	Not reported
CantonKessely 2024	Chad	Senegal	Prospective	Ballistic Medullar-Vertebral trauma	Mixed, 27	Multicenter/ International	Not reported
Fisk 1985	Tanzania		Retrospective	traumatic paraplegia	Mixed, 47	Academic	Not

							reported
Mhina 1993	Tanzania		Retrospective	spine fractures	Mixed, 54	Academic	Not reported
Loembe 1994	Gabon		Case Series	socio-economic difficulties in tuberculous spondylitis	Adult, 5	Academic	Not reported
Loembe 1994	Gabon					Academic	Not reported
Loembe 1994	Gabon		Retrospective	experience working in low resource hospital with appropriate medical and human structures	Adult, 22	Academic	Not reported
Levy 1998	Zimbabwe		Retrospective	traumatic spinal cord injuries	Adult, 136	Academic	Not reported
Gueye 1998	Senegal		Retrospective	epidemiology, management, and prognosis or PSCI	Mixed, 16	Academic	Not reported
Igun 1999	Nigeria		Retrospective	epidemiology, management, and outcome of spinal cord injuries	Mixed, 68	Academic	Not reported
Mwang'ombe 2000	Kenya		Retrospective	tumours associated with cord compression	Mixed, 38	Academic	Not reported
Solagberu 2002	Nigeria		Retrospective	etiology of spinal cord injury	Adult, 39	Academic	Not reported
Nwankwo 2003	Nigeria		Prospective	12-week rehabilitation programme for spinal cord injury	Adult, 74	Academic	Not reported
Andrews 2003	Ghana		Retrospective	elective non-instrumented ACDF	Adult, 41	Academic	Not reported
Ogunseyinde 2004	Nigeria		Retrospective	CT in the diagnosis of tuberculosis of the spine	Mixed, 22	Academic	Not reported
Gosselin 2005	Sierra Leone	United States, Italy	Retrospective	outcome of spinal injuries after returning to their community	Mixed, 24	Multicenter/ International	Not reported
Umaru 2005	Nigeria		Retrospective	pattern of spinal cord injury	Mixed, 36	Academic	Not reported
Olasode 2006	Nigeria		Retrospective	trends and challenges of spine surgery	Mixed, 463	Academic	Not reported
IbaBa 2006	Gabon		Cohort Study	outcome of isthmic and degenerative spondylolisthesis surgery	Adult, 21	Academic	Not reported
Singwe-Ngandeu	Cameroon		Cross-sectional	spectrum of rheumatic diseases	Mixed, 12494	Academic	Not

2007							reported
Njoku 2007	Nigeria		Combination of retrospective and prospective	management of complicated tuberculosis of the spine	Mixed, 92	Academic	Not reported
Nwadinigwe 2008	Nigeria		Retrospective	outcome of penetrating spinal cord injuries	Mixed, 22	Academic	Not reported
Obalum 2009	Nigeria		Retrospective	outcome of spinal cord injuries	Mixed, 468	Academic	Not reported
Emejulu 2009	Nigeria		Prospective	outcome of spinal cord injuries	Mixed, 62	Academic	Not reported
Kawu 2010	Nigeria		Retrospective	conservative treatment of closed thoracic and lumbar spine fractures following RTI	Mixed, 16	Academic	Not reported
Park 2010	Kenya	United States	Prospective	safety and efficacy of water-soluble contrast myelography for spinal disorders	40	Multicenter/ International	Not reported
Idowu 2011	Nigeria		Prospective	risk factors for pressure ulceration in traumatic spinal cord injury	Mixed, 105	Academic	Not reported
Mwachaka 2011	Kenya		Retrospective	clinical presentation and management of spinal tuberculosis	Mixed, 129	Academic	Not reported
Owolabi 2011	Nigeria		Prospective	clinical and radiological profile of non-traumatic paraplegia	Mixed, 98	Academic	Not reported
Kawu 2011	Nigeria	India	Prospective	cost of acute phase of injury in spinal cord injuries	Adult, 34	Multicenter/ International	Not reported
Ahidjo 2011	Nigeria		Prospective	predictor of mortality in patients with spinal cord injury	Mixed, 168	Academic	Not reported
Draulans 2011	Belgium		Retrospective	epidemiology of spinal cord injuries in Sub Saharan Africa	Mixed, 2926	Academic	Not reported
Kawu 2011	Nigeria	India	Retrospective	complications in patients with spinal cord injury	Adult, 422	Multicenter/ International	Not reported
Ogungbo 2011	Nigeria		Retrospective	surgical management of acute cervical spine injuries	Adult, 20	Not defined	Not reported
Iyun 2012	Nigeria		Prospective	presentation of pressure ulcers	Mixed, 67	Academic	Not reported
Ahidjo 2012	Nigeria	Nigeria	Prospective	predictor of mortality in spinal cord-injured patients	Mixed, 168	Multicenter/ International	Not reported

Kawu 2012	Nigeria		Retrospective	demographic associations in vertebral fractures and neurological deficit	Adult, 202	Academic	Not reported
Biluts 2012	Ethiopia		Cross-sectional	degenerative lumbar disc disease	Adult, 364	Academic	Not reported
Beavogui 2012							
Bayray 2012	Ethiopia	Ethiopia	Retrospective	osteoporotic fractures among adults admitted to zonal hospitals	Adult, 1169	Multicenter/ International	Yes
Rabiu 2016	Nigeria		Prospective	profile of neurosurgical admissions	Mixed, 331	Academic	No
Gandaho 2016	Benin Republic	Nigeria	Cross-sectional	evaluation attendance and affordaility at specialty center	Mixed, 2908	Multicenter/ International	Not reported
Debebe 2016	Ethiopia	United States, Canada		traumatic spinal cord injury victims	Mixed, 84	Multicenter/ International	Not reported
Nwankwo 2013	Nigeria		Retrospective	epidemiology and outcome of spinal cord injuries at three tertiary care centres	Mixed, 85	Academic	Not reported
Mezue 2013	Nigeria		Prospective	factors of transit injuries in spinal trauma patients	Mixed, 68	Academic	Not reported
Kpelao 2013	Senegal		Retrospective	epidemiology and outcome of spinal cord injuries at a tertiary care centres	Mixed, 68	Academic	Not reported
Adeolu 2013	Nigeria		Retrospective	indications and outcome of pedicle screws and rods for stablisation of the spinal column	Adult, 26	Academic	Not reported
Adeolu 2014	Nigeria		Retrospective	surgical management of upper cervical spine injuries	Adult, 12	Academic	Not reported
Nnadi 2014	Nigeria		Prospective	neurological recovery in traumatic spinal injury managed nonoperatively	Mixed, 76	Academic	Not reported
Doualla-Bija 2014	Cameroon		Cross-sectional	musculoskeletal features of multiple myeloma	Adult, 62	Not defined	Not reported
Papadopoulos 2015	West Africa	Greece, United States, Spain, Turkey	Retrospective	severe rigid kyphosis	Mixed, 45	Multicenter/ International	Not reported
Lofvenmark 2015	Botswana	Sweden	Retrospective	epidemiology of traumatic spinal cord injuries in road traffic crashes	Mixed, 49	Multicenter/ International	Yes
Uche 2015	Nigeria		Retrospective	risk factors for poor outcome in cervical spine injury	Mixed, 195	Academic	Not reported
Lehre 2015	Ethiopia	Norway, Cambodia	Retrospective	epidemiology and outcome after	Mixed, 146	Multicenter/	Not

				surgical treatment for spinal injuries		International	reported
Diomande 2015	Abidjan		Retrospective	epidemiological, clinical and etiological characteristics of non-traumatic osteoarticular disorders	Adult, 157	Academic	Not reported
Biluts 2015	Ethiopia		Cross-sectional	local hospital patterns of spinal injury	Mixed, 385	Academic	Not reported
Fidele 2016	Ethiopia	Rwanda	Retrospective	etiology, clinical presentation and outcome of nontraumatic myelopathies	Mixed, 105	Multicenter/ International	Not reported
Park 2016	Kenya		Retrospective	efficacy of surgical approach	Mixed, 450	Academic	Not reported
Nkusi 2016	Rwanda		Prospective	cause of missed or delayed diagnosis of cervical spine injuries	Adult, 42	Academic	Not reported
Ametefe 2016	Ghana		Retrospective	occurrence and patterns of spine injury, treatment and outcomes	Mixed, 185	Academic	No
Uche 2016	Nigeria		Retrospective	complications and outcome of skull traction devices for cervical spine injury	Mixed, 105	Academic	No
Ojo 2016	Nigeria		Cross-sectional	SSI in posterior spine surgery	Adult, 62	Academic	No
Rashid 2017	Tanzania	Netherlands	Retrospective	epidemiology of traumatic spinal cord injury	Mixed, 125	Multicenter/ International	Not reported
Musubire 2017	Uganda	United States, Burundi	Retrospective	etiologies of non-traumatic myelopathy	Mixed, 12116	Multicenter/ International	Yes
Nasidi 2019	Nigeria		Cross-sectional	quality of life of persons with spinal cord injury	Adult, 81	Academic	Not reported
Paulus-Mokgachane 2019	Botswana	South Africa	Cross-sectional	access to primary care services for patients with spinal cord injuries	Adult, 57	Multicenter/ International	No
Lofvenmark 2017	Botswana	Sweden	Prospective	clinical and functional outcomes 2 years after traumatic spinal cord injury	Adult, 38	Multicenter/ International	Yes
Lofvenmark 2017	Botswana	Sweden	Prospective	clinical and functional outcomes after traumatic spinal cord injuries	Mixed, 39	Multicenter/ International	Yes
Ekechukwu 2017	Nigeria	Benin, Azare	Prospective	predictors of quality of life among persons with spinal cord injury	Mixed, 64	Multicenter/ International	No
Bjerke 2017	Ghana	United States	Prospective	precipitating factors for neurologic injury and prognosis	Mixed, 88	Multicenter/ International	Not reported
Bemora 2017	Madagascar		Retrospective	frequency and epidemiology of spinal	Adult, 139	Academic	Not

				cord trauma			reported
Kitya 2017	Uganda	United States	Prospective	reliability, effectiveness and safety of conventional myelography	51	Multicenter/ International	Not reported
Stothers 2017	Uganda	Canada, South Africa	Cross-sectional	knowledge of spinal cord injury and priorities for quality of life	Mixed, 283	Multicenter/ International	Yes
Rabiu 2017	Nigeria		Prospective	clinical outcomes for patients managed using the Adeolu technique	Adult, 18	Academic	Not reported
Eaton 2017	Malawi	United States	Retrospective	mortality in trauma patients based on anatomic location of injury	Adult, 8569	Multicenter/ International	Yes
Moshi 2017	Tanzania	Sweden	Retrospective	incidence, etiology and clinical outcomes of traumatic spinal cord injury	Mixed, 288	Multicenter/ International	Yes
Fall 2017	Senegal		Retrospective	diagnostic and evolutionary features of myeloma patients	Adult, 136	Academic	Not reported
Ojo 2017	Nigeria	United States	Cross-sectional	spinal cord decompression with or without spinal stabilization	Adult, 35	Multicenter/ International	No
Ekeke 2017	Nigeria		Retrospective	trauma-related deaths	Mixed, 527	Academic	Not reported
Brown 2018	Zambia	USA	Cohort Study	prevalence of neurologic disorders in the ICU	Mixed, 33	Multicenter/ International	Not reported
Kabenkama 2018	Democratic Republic of the Congo		Prospective	vertebral fractures in postmenopausal women	Adult, 430	Academic	Not reported
Admasu 2018	Ethiopia		Retrospective	subaxial spinal injury treated with open surgical fixation	85	Academic	Not reported
Renaud 2018	Madagascar	United Kingdom	Prospective	specialist rehabilitation service for spinal cord injury	Mixed, 50	Multicenter/ International	Yes
Atobatele 2018	Nigeria	United Kingdom	Prospective	community reintegration and clinical/psychosocial attributes in spinal cord injury	Adult, 50	Multicenter/ International	Not reported
Gbadamosi 2018	Ghana		Cross-sectional	Magnetic Resonance Imaging features of patients with Non-Traumatic Spinal Cord Injury	Mixed, 141	Academic	Not reported
Gaye 2019	Senegal	Mauritania	Retrospective	characteristics of NMO spectrum disorders	Mixed, 16	Multicenter/ International	Not reported
Eaton 2019	Malawi	USA	Prospective	epidemiology and outcomes on traumatic spinal cord injury	Adult, 46	Multicenter/ International	Yes

Musubire 2019	Uganda	United States	Prospective	presentation, MRI patterns, and survival among subjects with non-traumatic spinal cord injury	Adult, 103	Multicenter/ International	Yes
Adeolu 2019	Nigeria		Prospective	effectiveness of closed reduction of cervical spine injuries using cervical traction	Mixed, 74	Academic	Not reported
Davey 2019	Tanzania	USA	Prospective	socioeconomic impact of musculoskeletal injury	Mixed, 200	Multicenter/ International	No
Leidinger 2019	Tanzania	United States	Retrospective	demographics, management, costs, and outcomes of spine trauma	Adult, 180	Multicenter/ International	Not reported
Balarabe 2019	Nigeria		Retrospective	frequency of neurologic disorders	Mixed, 2537	Academic	No
Divanoglou 2019	Botswana	Iceland, Australia, Sweden, Poland	Prospective	effects of the Active Rehabilitation for individuals with spinal cord injury	Adult, 19	Multicenter/ International	Not reported
Bellet 2019	Tanzania	Netherlands	Retrospective	sociodemographic and clinical characteristics of cervical Traumatic Spinal Cord Injuries	Mixed, 105	Multicenter/ International	Not reported
Holmes 2019	Nigeria	USA	Retrospective	characteristics and risk factors of traumatic spinal injury	Mixed, 3025	Multicenter/ International	Not reported
Lessing 2020	Tanzania	United States, Spain	Retrospective	describe population and outcomes in operative vs non-operative patients	Mixed, 270	Multicenter/ International	Not reported
Lessing 2020	Tanzania	United States, Spain			Mixed, 267	Multicenter/ International	Not reported
Adedigba 2020	Nigeria	USA, Canada	Prospective	nonoperative management in degenerative spinal disease after traumatic spinal cord injury	Adult, 28	Multicenter/ International	Not reported
Nade 2020	Tanzania		Cross-sectional	challenges faced by individuals with SCI during Clean Intermittent Catheterisation	Mixed, 48	Academic	Yes
Moshi 2020	Tanzania	South Africa	Prospective	injury mechanism for spinal cord injury involving ox-carts	Adult, 163	Multicenter/ International	Not reported
Moshi 2021	Tanzania	Sweden	Cross-sectional	quality of life of persons with traumatic spinal cord injury	Mixed, 80	Multicenter/ International	Yes
Anthony 2021	Africa	United Kingdom, Saudi Arabia, Ethiopia, Ghana	Retrospective	prevalence and incidence of pressure ulcers	Mixed, 6846	Multicenter/ International	Yes
Toluse 2021	Nigeria		Retrospective	epidemiological characteristics of traumatic spinal cord injury	Mixed, 99	Academic	Not reported

Ishaku 2021	Nigeria		Retrospective	pattern and outcome of traumatic spinal cord injury	Adult, 81	Academic	No
Moshi 2021	Tanzania	Sweden	Prospective	traumatic spinal cord injury and clinical complications	Adult, 87	Multicenter/ International	Yes
Balcha 2021	Ethiopia		Retrospective	cervical spine Magnetic Resonance Imaging patterns	Adult, 160	Academic	Not reported
Ikwuegbuenyi 2022	Africa		Cohort Study	barriers to seeking care to use neurosurgical services	Adult, 652	Not defined	No
Kpelao 2022	Togo		Retrospective	etiology and outcome of spinal cord compression	Mixed, 113	Academic	Not reported
Atenguena 2022	Cameroon		Retrospective	pattern of metastatic spinal cord compression	Adult, 151	Academic	Not reported
Gulamhussein 2023	Tanzania	USA	Prospective	out-of-pocket costs for injured patients	Adult, 355	Multicenter/ International	No
Leidinger 2023	Tanzania	Spain, United States, Kenya	Retrospective	decision-making and in-hospital mortality among patients with acute spinal trauma	Adult, 284	Multicenter/ International	Not reported
Shu 2023	Cameroon		Retrospective	etiology, management principles, and prognosis of traumatic lumbar spine injury	Mixed, 70	Academic	No
Waterkeyn 2023	Tanzania	Belgium, United States	Retrospective	characteristics of traumatic spinal injuries from coconut tree falls	Mixed, 44	Multicenter/ International	Not reported
Alihonou 2020	Benin	Benin	Retrospective	management of neurosurgical emergencies	Mixed, 397	Multicenter/ International	Not reported
Magogo 2021	Tanzania	United States	Retrospective	operative decision-making process, implant availability and surgical judgment	Mixed, 97	Multicenter/ International	No
Eisner 2021	United States	USA	Scoping Review	training and resources for prehospital traumatic spinal cord injury		Multicenter/ International	Not reported
Zuckerman 2021	Tanzania	United States, Spain	Retrospective	presentation and operative treatment patterns, predictors of neurologic improvement	Mixed, 101	Multicenter/ International	Not reported
Lessing 2022	Tanzania	United States, Spain	Retrospective	cost-effectiveness analysis of operative treatment of traumatic spinal injury	Mixed, 270	Multicenter/ International	No
Ashipala 2022	Namibia		Prospective	experiences with spinal cord injury admitted to the rehabilitation unit	Adult, 15	Academic	No

Admasu 2023	Ethiopia		Cross-sectional	post op neurologic status in incomplete thoracic and lumbar spine injuries	Mixed, 64	Academic	Not reported
Alfin 2023	Nigeria		Retrospective	clinical profile and determinants in patients with traumatic spinal cord injury	Adult, 296	Academic	No
Oyemolade 2023	Nigeria		Prospective	clinical epidemiology of pediatric traumatic spinal cord injury	Pediatric, 20	Academic	Not reported
Jesuyajolu 2023	Nigeria		Retrospective	traumatic spinal cord injuries	Mixed, 1332	Not defined	Not reported
